# Genome-Wide Identification and Molecular Characterization of the Growth-Regulating Factors-Interacting Factor Gene Family in Tomato

**DOI:** 10.3390/genes11121435

**Published:** 2020-11-28

**Authors:** Guo Ai, Dedi Zhang, Rong Huang, Shiqi Zhang, Wangfang Li, John K. Ahiakpa, Junhong Zhang

**Affiliations:** 1 Key Laboratory of Horticultural Plant Biology, Ministry of Education, Huazhong Agricultural University, 430070 Wuhan, China; aiguo2009@webmail.hzau.edu.cn (G.A.); 15207163154@163.com (D.Z.); 13934762841@163.com (R.H.); 15927360163@163.com (S.Z.); liwangfang_hzau@163.com (W.L.); jonahiakpa@outlook.com (J.K.A.); 2 Key Laboratory of Urban Agriculture in Central China, Ministry of Agriculture and Rural Affairs, 430070 Wuhan, China

**Keywords:** GIF, GRF, *Solanum lycopersicum*, transcriptional co-activator, organ size

## Abstract

Growth-regulating factors-interacting factor (GIF) proteins play crucial roles in the regulation of plant growth and development. However, the molecular mechanism of GIF proteins in tomato is poorly understood. Here, four *SlGIF* genes (named *SlGRF1a*, *SlGIF1b*, *SlGIF2*, and *SlGIF3*) were identified from the tomato genome and clustered into two major clades by phylogenetic analysis. The gene structure and motif pattern analyses showed similar exon/intron patterns and motif organizations in all the *SlGIFs*. We identified 33 *cis*-acting regulatory elements (CAREs) in the promoter regions of the *SlGIFs*. The expression profiling revealed the four *GIFs* are expressed in various tissues and stages of fruit development and induced by phytohormones (IAA and GA). The subcellular localization assays showed all four GIFs were located in nucleus. The yeast two-hybrid assay indicated various growth-regulating factors (SlGRFs) proteins interacted with the four SlGIF proteins. However, SlGRF4 was a common interactor with the SlGIF proteins. Moreover, a higher co-expression relationship was shown between three *SlGIF* genes and five *SlGRF* genes. The protein association network analysis found a chromodomain helicase DNA-binding protein (CHD) and an actin-like protein to be associated with the four SlGIF proteins. Overall, these results will improve our understanding of the potential functions of *GIF* genes and act as a base for further functional studies on *GIFs* in tomato growth and development.

## 1. Introduction

Transcription factors (TFs) are a class of proteins, which are regulators of transcription of target genes, and play essential roles in various processes of growth and development in plants [1,2]. TFs mediate expression of target genes by binding to their promoters [3,4,5,6]. Growth-regulating factors (GRFs) belong to a class of plant-specific TFs factors involved in the regulation of stem, leaf development, flower and seed formation, root development, growth processes, and response to stress [7,8,9,10,11,12,13]. Growth-regulating factors-interacting factors (GIFs) predominantly function as transcription co-activators of their interaction proteins, GRFs [7,8,9].

GIFs are a class of transcriptional activators, interacting with GRFs to form functionally transcriptional complexes [7,14,15,16]. The first member of the GIF family identified was AtGIF1 and used as a bait in a yeast two-hybrid assay [14]. AtGIF1 functions as a transcriptional co-activator, involved in the control of leaf growth and morphology [14]. It also encodes a homolog of the human synovial sarcoma translocation protein (SYT), one important transcription co-activator [14]. In *Arabidopsis thaliana* (*A. thaliana*), the *GIF* gene family contains three proteins, GIF1, GIF2, and GIF3, and play essential roles in vegetative and reproductive organs development [17,18]. Engineered *gif1* mutants involving AtGIF1 (also known as ANGUSTIFOLIA3 (AN3)) result in a decreased cell number with narrow-leaf phenotypes, and enhanced AtGIF1 expression levels leads to increased leaf areas by increasing cell numbers in leaf primordial [19]. Interestingly, an *an3* mutant involving AN3 exhibits a reduced cell number, but excessively enlarged cells [20]. GIF1 is also reported to be synthesized in mesophyll cells and transported into epidermal cells to regulate the proliferation of both epidermal and mesophyll cells in leaves [21]. Additionally, AtGIF1 is involved in the establishment of cotyledon identity by suppressing ectopic root formation [22] and functions in adaxial/abaxial patterning and leaf growth [23]. 

In Arabidopsis, AtGIF1 interacts with AtGRF1, AtGRF2, AtGRF3, AtGRF4, AtGRF5, and AtGRF9 [14,19,24]. GIF1 affects leaf development and cell proliferation by interacting with AtGRF3 [24] and AtGRF5 [19], respectively. In rice, GIFs are involved in OsGRF4 regulation of grain size and yield [10,25]. Studies show that GIF1 also binds to a SWI/SNF chromatin remodeling complex to regulate the transcription of downstream genes [26]. Moreover, the function of the GIF1-associated SWI/SNF chromatin remodeling complex is conserved between dicots and monocots. The transcription of GRF1 and GRF10 facilitates binding with AtGIF1/AN3 in cell division and expansion which contribute to leaf growth [27]. GIF1 binds to the promoter of *unranched3* (*ub3*), the inflorescence architecture gene, and regulates the expression of several genes involved in shoot architecture and meristem in maize [28]. GIFs function in maintaining precise expression patterns of key developmental regulators, while GIFs/AN3 complexes bind directly to the promoters of PLETHORA1 (PLT1) and SCARECROW (SCR) to fine-tune a quiescent center (QC) and root meristem during root development [29]. Recently, *GIF1* was found to be the direct downstream target gene of the KIX-PPD-MYC complex in regulating seed size [30].

Tomato is an important vegetable farmed globally for essential nutrients and minerals and for industrial processing into tomato paste [31,32,33]. The functions of *GIF* genes in tomatoes remain unclear at present. In this study, we identified and characterized four tomato *GIF* genes, including their phylogenetic relationships, *cis*-acting regulatory elements (CAREs), subcellular localization, expression profiles in various tissues at varied growth stages, expression patterns in response to phytohormones (GA, IAA, and breaker (BR)), protein–protein interactions between GIFs and GRFs, co-expression relationships between *GIFs* and *GRFs*, and *SlGIF* genes association networks. These results provide a theoretical basis for further functional studies of SlGIF proteins in tomatoes.

## 2. Materials and Methods 

### 2.1. Identification of Tomato GIF Genes

To identify *GIF* genes in tomatoes, the SSXT domain of the AtGIF1 protein were used as seed sequences to search the National Center for Biotechnology Information (NCBI https://www.ncbi.nlm.nih.gov/) and Sol Genomics Network (SGN https://solgenomics.net/) databases through BLASTP. To ensure all putative *SlGIF* genes were included, the protein sequences of the identified *SlGIFs* were further confirmed through the Phytozome website (http://www.phytozome.net/) and the Plaza website (http://bioinformatics.psb.ugent.be/plaza/) databases. We used the ProtParam database (https://web.expasy.org/protparam/) to assess their physio-chemical characteristics (molecular weight and isoelectric point).

We predicted the conserved motifs of the GIF proteins using the Online Conserve Domain server (https://www.ncbi.nlm.nih.gov/Structure/cdd/wrpsb.cgi). The *GIF* gene structure was visualized using the GeneDoc software based on the primary sequence information obtained from the SGN database.

### 2.2. Phylogenetic Analyses

Multiple alignment of all the *GIFs* proteins was performed using ClustalX [34], and phylogenetic tree was constructed by MEGA (version 6) [35] with a bootstrap of 1000 replicates using the neighbor-joining (NJ) method. 

### 2.3. Identification of CAREs in the Promoter

Sequences from the promoter region (about 3 kb upstream of the start codon) of each gene was retrieved from the SGN database (https://solgenomics.net/organism/genome) in Generic File Format (GFF) to identify putative CAREs using the PlantCare database (http://bioinformatics.psb.ugent.be/webtools/plantcare/html/). The identified CAREs visualized using the Toolkit for Biologists integrating various biological data handling tools (TBtools) [36].

### 2.4. Plant Materials and Hormone Treatment

A *Solanum lycopersicum* cultivar, Alisa Craig, was used in this study. The seeds were germinated in 50-hole flats in the soil and grown in a greenhouse with a 16 h light and 8 h night photoperiod. Two-leaf-stage tomato seedlings were transplanted to 10 cm × 10 cm × 10 cm compost plastic pots and grown in a common greenhouse. Six-leaf tomato seedlings with a similar growth were chosen for the plant hormones treatment to check the expression of genes. The seedlings were sprayed with 100 μM GA, 100 μM IAA, and 100 μM BR for the hormone treatment. The seedlings treated with water were as a control. The leaves were collected after 0, 0.5, 1, 2, 4, 8, 12, and 24 h, and all the samples were frozen in liquid nitrogen and stored in –80 °C. Three biological samples in each process were obtained for the following experiments.

### 2.5. RNA Extraction and Reverse Transcription Polymerase Chain Reaction (RT-PCR) Analysis

Total RNA was extracted using TRIzol reagent (Aidlab Biotechnologies, Beijing, China;). A 3 μg sample of RNA was reversely transcribed into complementary DNA (cDNA) using a HisScript II 1st Strand cDNA Synthesis Kit (Vazyme, Nanjing, China;). RT-PCR was performed to determine the transcript levels of target genes using 384-well blocks with QuantStudio (TM) 6 Flex System (ThermoFisher Scientific; Waltham, MA, USA). Three technical replicates were performed, and each replicate of 10 μL reaction containing 5 μL SYBR mix, 4.2 μL cDNA sample, and 0.4 μL of 10 μM gene-specific primes went through the following amplification process: a 3 min pre-incubation step at 95 °C, followed by 40 cycles of 95 °C for 30 s, 58 °C for 15 s, and 72 °C for 20 s. The comparative 2^−ΔΔCт^ method was used to calculate the relative levels of target gene expressions [37], and the β-actin gene (Soly11g008430) was used as an internal control. The primers for RT-PCR are listed in Table S1.

### 2.6. Subcellular Localization 

The full-length coding regions without a stop codon of each *GIF* genes were amplified by PCR using gene-specific primers containing homologous recombination and introduced into a yellow fluorescent protein (YFP) vector to generate a construct using the ClonExpress II One Step Cloning Kit (Vazyme, Nanjing, Jiangsu China). Four-week-old leaves of *Nicotiana tabacum* were used to perform a transient expression assay mediated by *Argobacterium tumefaciens* strain (GV2260) carrying GIF-GFP fusion proteins and GV2260 carrying the nucleus and cytoplasm marker 35S:RFP as previously described [38]. The tobacco leaves were used for YFP and RFP fluorescence signal observation using a Leica confocal microscope (LeicaSP8). The primers for subcellular localization assays are listed in Table S1.

### 2.7. Yeast Two-Hybrid Assay

The full-length coding regions of the *GIF* genes were amplified by PCR using gene-specific primers containing homologous recombination sites and were cloned into the bait vector pGBKT7. The full-length coding regions of the GRF genes were cloned into the prey vector pGADT7 by gene-specific primers containing homologous recombination sites. Each pair of bait–prey vectors was co-transformed into the yeast strain AH109 following the instructions of Matchmaker Gold Two-hybrid System (Clontech, Mountain View, CA, USA). The transformed yeasts were plated on an SD medium lacking leucine and tryptophan (SD/-Trp-Leu). After the yeast cells grew at 30 °C for 3–4 days, colonies were picked and transferred to an SD medium lacking leucine, histidine, adenine, and tryptophan (SD/-Trp-Leu-His-Ade). The yeast concentrations were estimated by measuring their optical densities at 600 nm. These were maintained at the same concentration (OD_600_:1) for protein interactions assay. The strength of the interaction depended on the yeast growth conditions [19]. The combination of SlGIFs introduced into the pGBKT7 vector and the empty pGADT7 vector were used as negative controls, as well as the combination of empty pGBKT7 and SlGRFs introduced in the pGADT7 vector. pGBKT7-53 and pGADT7-RceT were used as positive controls The primers for the yeast two-hybrid assays are presented in in Table S1.

### 2.8. Expression Profiles and the Correlation Coefficients Analysis

The RNA-Seq data of different tissues at various developmental stages of the fruit of the tomato cultivar, Heinz 1706, were accessed from the Tomato Expression Atlas database (TEA). Tissues including root, leaf, flower, flower bud, fruit at different sizes (1, 2, and 3 cm), mature green fruit, BR fruit, and fruit at 10 days after breaker were retrieved from the TEA database. In addition, the expression data for leaf, immature green fruit, BR fruit and fruit at 5 days after breaker were accessed from LA1589 (*Solanum pimpinellifolium*) [32,39]. The normalized expressions (RPKM) of *SlGIFs* were downloaded from the supplementary files [32]. 

The expression profiles of SlGIFs and SlGRFs from the RNA-seq in 536 samples from 18 transcriptome assays are listed in Table S4. The RPKM of *SlGRFs* and *SlGIFs* were accessed from the Tomato Functional Genomic database (http://ted.bti.cornell.edu/cgi-bin/TFGD/digital/home.cgi). The correlation coefficients between *SlGRFs* and *SlGIFs* were computed using the R language (R version 3.6.3) (https://www.r-project.org/).

## 3. Results

### 3.1. Identification of GIF Genes in Tomatoes

In this study, four *GIF* gene members were identified in the tomato genome (Table 1). To further understand SlGIF proteins, the amino acid (aa) length, the chromosome location, the molecular weight (Mw), and the theoretical isoelectric points (pI) of the four SlGIF proteins were analyzed (Table 1). The *SlGIF* genes were distributed on four chromosomes (chromosomes 3, 4, 10, and 11). The lengths of SlGIF proteins varied from 199 aa residues (SlGIF2) to 222 aa residues (SlGIF1b), with the Mw ranging from 21.74 kDa (SlGIF3) to 23.59 kDa (SlGIF1b). The pI varied from 5.85 (SlGIF2) to 6.60 (SlGIF1a).

### 3.2. Phylogenetic Analysis of the SlGIF Family Genes

**Figure 1:**
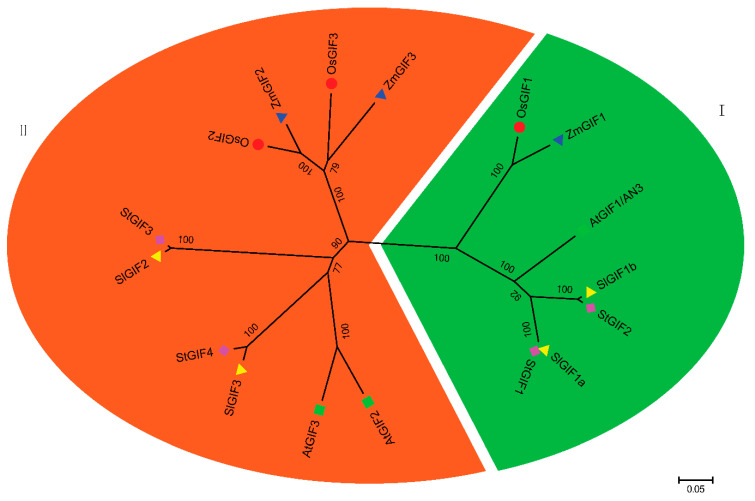
Phylogenetic relationship of GIF proteins. The unrooted tree was constructed using MEGA6 through the neighbor-joining method at 1000 bootstrap replicates based on the alignment of GIF protein sequences in Arabidopsis, rice, maize, potatoes, and tomatoes. The different markers before protein names stand for different plants. All these GIF proteins were clustered into two clades (**I** **and II**) in the phylogenetic tree.

A phylogenetic tree of the *GIF* genes from five species was constructed to study their evolutionary patterns in the plant kingdom. SlGIFs and their counterparts in Arabidopsis, rice, maize, and potatoes were used for the phylogenetic analysis. The unrooted phylogenetic tree was constructed after the alignments of the full-length GIFs protein sequences from the five species containing three GIF proteins each in *A. thaliana*, *Oryza sativa* (*O. sativa*), and *Zea mays* (*Z. mays*) and four GIF proteins each in *Solanum tuberosum* and *Solanum lycopersicum* (Table S2). All four tomato GIFs proteins showed high similarity with AtGIF1/AN3 and were named SlGIF1a, SlGIF1b, SlGIF2, and SlGIF3 according to their sequence similarity to GIFs in Arabidopsis (Figure 1). The four SlGIF proteins were clustered into two clades (I and II) in the phylogenetic tree (Figure 1). Clade I contained SlGIF1a and SlGIF1b, while clade II contained SlGIF2 and SlGIF3.

### 3.3. Gene Structures, Conserved Domains, and CAREs in the Promoters of SlGIF Genes in Tomatoes

**Figure 2:**
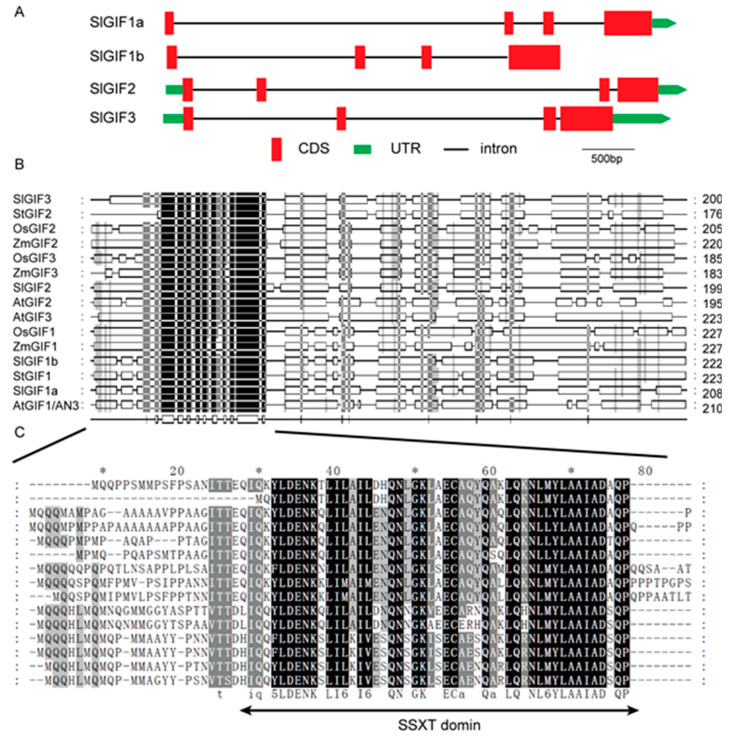
Structure and conserved motif analysis of the *GIF* genes. (**A**) The exon and intron structures of the tomato *GIF* genes. The gene structures of the *SlGIF* genes were illustrated using the Gene Structure Display Server (http://gsds.cbi.pku.edu.cn/) and the IBS software [41]. The red box and the green box represent the CDS and UTR, respectively. The solid line stands for the intron. (**B**) Motif analysis of the SlGIF proteins. The conserved motifs of all GIF proteins in this study were identified using ClustalX and GeneDoc software. (**C**) Detailed SSXT domains from the GIF proteins.

To further study the potential functions of the *GIF* genes, the structures of the *GIF* gene sequences were analyzed using the PlantCare database [40]. Each of the four *SlGIF* genes contained four exons and three introns (Figure 2A). The lengths of all introns were longer than those of all the exons (Figure 2A). The N-terminal regions of the GIF proteins contained the conserved domain, SSXT (Figure 2B), which is involved in synovial sarcoma in humans [14]. The conserved domain contained the motif “LDENK*LI*I*QN*GK *EC*Q*LQ**NL*YLAAIAD*QP” (Figure 2). 

**Figure 3:**
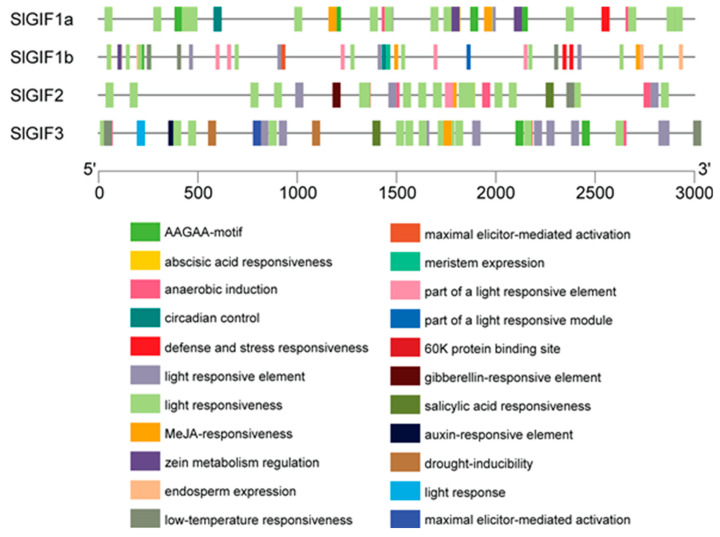
Identified *cis*-elements in the promoters of the *SlGIF* genes. The line represents the upstream of the *GIF* genes. Different colored rectangulars represent different *cis*-elements. The lengths of the promoters of *SlGIF* genes are 3 kb.

The *CAREs* in the promoter sequences play essential roles in gene transcription. Therefore, characterizing them in the promoter of *SlGIF* genes in tomatoes may provide insights into the functions of *SlGIF* genes. A total of 33 CAREs with predicted functions were identified from the promoters of the four *SlGIF* genes (Table 2). Among the 33 CAREs (Figure 3), six (ABER, CAAT-box, G-box, TATA-box, TCA-element, and ARE) were common to all four *SlGIF* genes. TATA-box and CAAT-box were the most common CAREs. G-box was involved in light responsiveness, implying the functions of GIF proteins may be influenced by light. AERE and TCA-elements were responsive to abscisic acid (ABA) and salicylic acid (SA), indicating that GIFs may play an important role in ABA and SA response. The remaining *CAREs* were divided into five groups, containing growth-, metabolism-, hormone-, stress-, and light-responsive elements (Figure 3). Phytohormone-responsive elements included auxin-responsive elements (AuxRR-core, TGA-element), MeJA-responsiveness (CGTCA-motif and TGACG-motif), and gibberellin-responsiveness (TATC-element). Interestingly, CAREs involved in circadian controls were found in *SlGIF1a* and *SlGIF1b* promoters, signifying their potential functions may be influenced by day length. This is consistent with the function of AN3 in modulating light-induced root elongation [5], as shown by the clustering of GIFs in clades I and II (Figure 3).

### 3.4. Expression Patterns of the SlGIFs in Different Tomato Tissues

**Figure 4:**
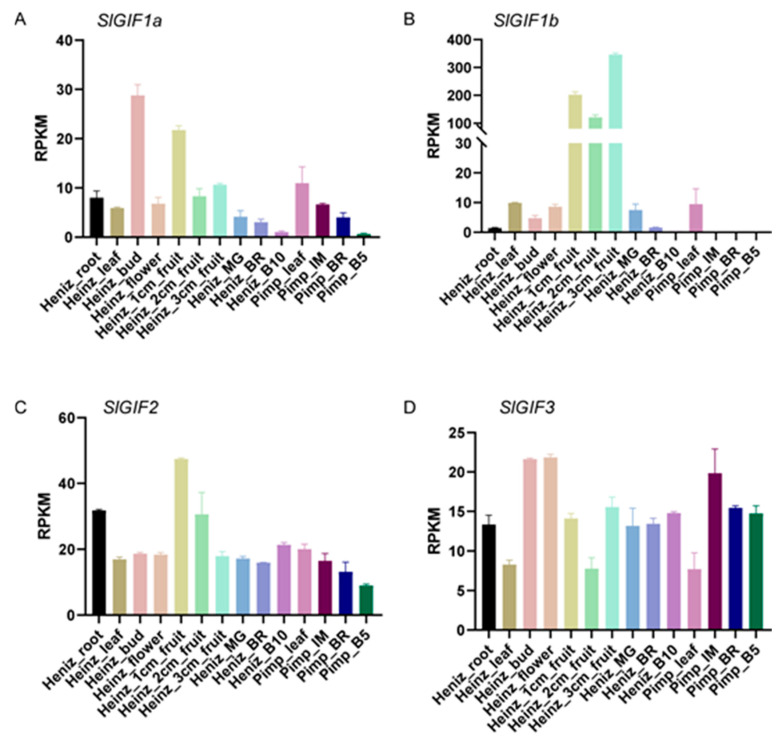
Expression profiles of the *SlGIF* genes (*SlGIF1* (**A**), *SlGIF2* (**B**), *SlGIF3* (**C**), and *SlGIF4* (**D**)) in different tissues from Heinz 1706 and LA1589. Normalized expressions (RPKM) of each genes in different tissues, containing root, leaf, bud, flower, 1 cm fruit, 2 cm fruit, 3 cm fruit, mature green (MG), breaker (BR, early ripening), and 10 days post breaker (B10, red ripe) from Heinz 1706 and leaf, immature fruit (IM), breaker (BR), and red (B5) from *Solanum pimpinellifolium (S. pimpinellifolium*) (Pimp) LA1589 [32] stand for the expression of *SlGIF* genes in different tissues. RPKM are displayed as means ± SD (*n* = 2).

Based on the important functions of the GIF proteins in various growth processes in plants, we studied their expression patterns in different tomato tissues. The four *GIFs* were expressed in the various tissues in Heinz 1706 (Figure 4). The expression levels of *SlGIF2* and *SlGIF3* showed similar patterns in immature green fruit, mature green fruit, BR fruit, and red ripe fruit, but the expression of *SlGIF1a* exhibited higher relative expression in immature green fruit (1, 2, and 3 cm) and declined sharply in the late developmental stages of ripening (Figure 4). The expressions of *SlGIF1a* and *SlGIF1b* shared a similar expression pattern, but the expression levels of *SlGIF1b* were significantly higher in immature green fruit than those of *SlGIF1a*. In short, the expression of *SlGIF1a* was relatively higher in immature green fruit and decreased during ripening. The expression of *SlGIF1b* was low in root, leaf, bud, and flower, but it maintained higher relative expression in immature green fruit. The transcript levels of *SlGIF2* recorded relatively high expression in root, 1 cm fruit, and 2 cm fruit but were lowly expressed in other tissues/stages. The expression levels of *SlGIF3* were lower in leaf and 2 cm fruit stages and were relatively higher in root, bud, flower, 1 cm fruit, 3 cm fruit, BR and red ripe fruit stages. However, the expression patterns of the *GIF* genes in LA1589 were different from in Heinz 1706. *SlGIF1*a showed a relatively lower expression in immature green fruit, BR, and fruit at 5 days after breaker, and the expressions of *SlGIFb* were hardly be detected in IM, BR, and red ripe stages in LA1589. The expression levels of *SlGIF2* and *SlGIF3* showed similar patterns among the four tissues/stages in LA1589. In summary, the different expression levels of the *GIF* genes between Heinz 1706 and LA1589 indicated functional divergence in domesticated and wild tomatoes.

### 3.5. Expression Profiles of the SlGIF Genes under Phytohormone Treatments

**Figure 5:**
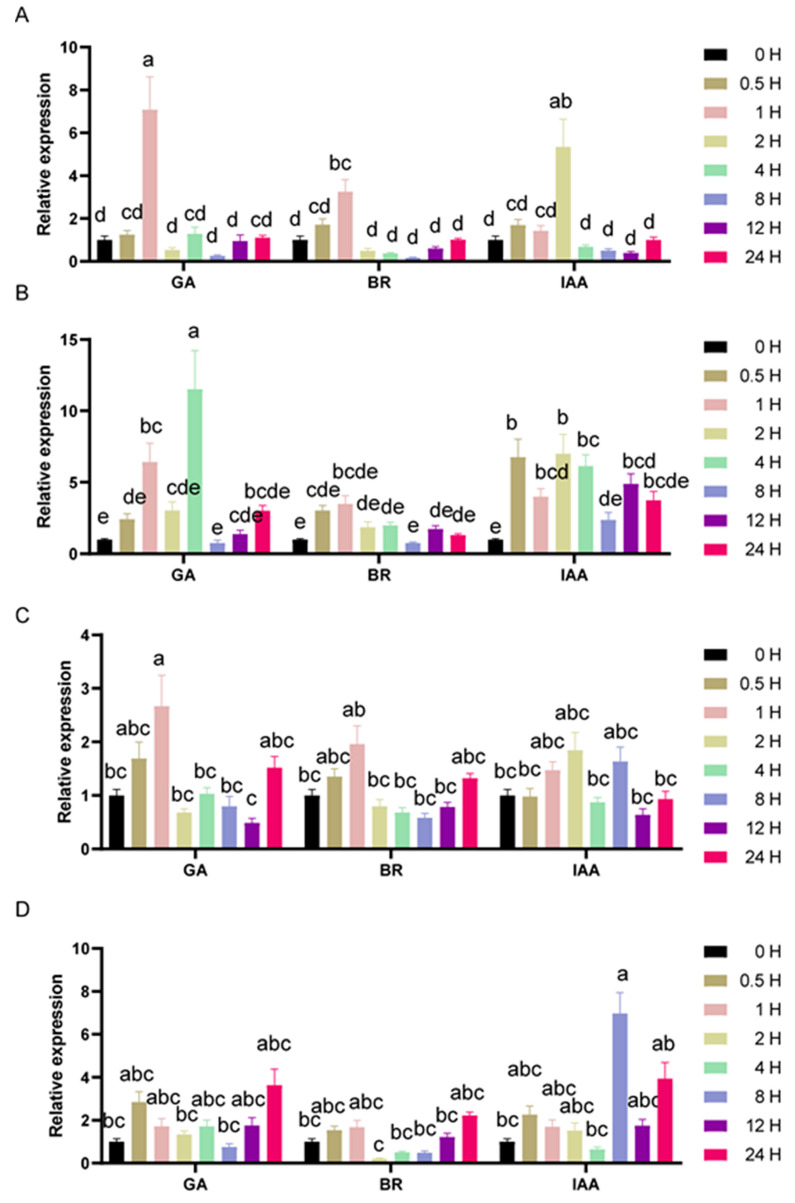
Expression profiles of *SlGIF* genes (*GIF1a* (**A**), *GIF1b* (**B**), *GIF2* (**C**), and *GIF3* (**D**)) under BR, GA, and IAA treatments. The numbers 0 H, 0.5 H, 1 H, 2 H, 4 H, 8 H, 12 H, and 24 H indicate the time after the treatment. The expressions of the treated plants were compared with those of the untreated plants after the normalization of values with an internal reference. The error bars represent the standard errors among three independent replicates, and the different letters above the bars indicate statistically significant differences at a 5% level of significance according to Tukey’s pairwise comparison tests.

Phytohormones play essential roles in the coordination of growth and development under various environmental conditions. BRs are a class of steroid phytohormones, known for their functions in cell division and elongation [42,43,44,45]. Similarly, GA and IAA have also been functionally implicated in cell proliferation and expansion in tomatoes [46]. Functional studies showed that GIFs play essential roles in cell proliferation and expansion in Arabidopsis [7,14,20,24]. These hormones were chosen to check their effects on GIFs responses and expressions. We analyzed the expression profiles of the *SlGIFs* genes under phytohormone treatments with BR, GA, and IAA. Four *SlGIF* genes were induced by GA and IAA treatments, especially for *SlGIF1a* and *SlGIF1b* (Figure 5). They were less sensitive to BR treatments. Among the four *SlGIF* genes, *SlGlF1b* showed consistently lower relative expression levels after the BR treatment compared to GA and IAA. However, *SlGIF1a* sharply decreased in expression after peaking under the GA and IAA treatments, whereas the expression of *SlGIF3* gradually decreased after peaking under the IAA and GA treatments. The expression levels of *SlGIF1b* and *SlGIF2* decreased slowly after the peak under the IAA treatment. The expression levels of *SlGIF1b* and *SlGIF2* were downregulated sharply after the peak at 4 and 1 h under the GA treatment, respectively. This suggested different roles of *SlGIFs* in growth signals. 

### 3.6. Subcellular Localization of the SlGIF Proteins

**Figure 6:**
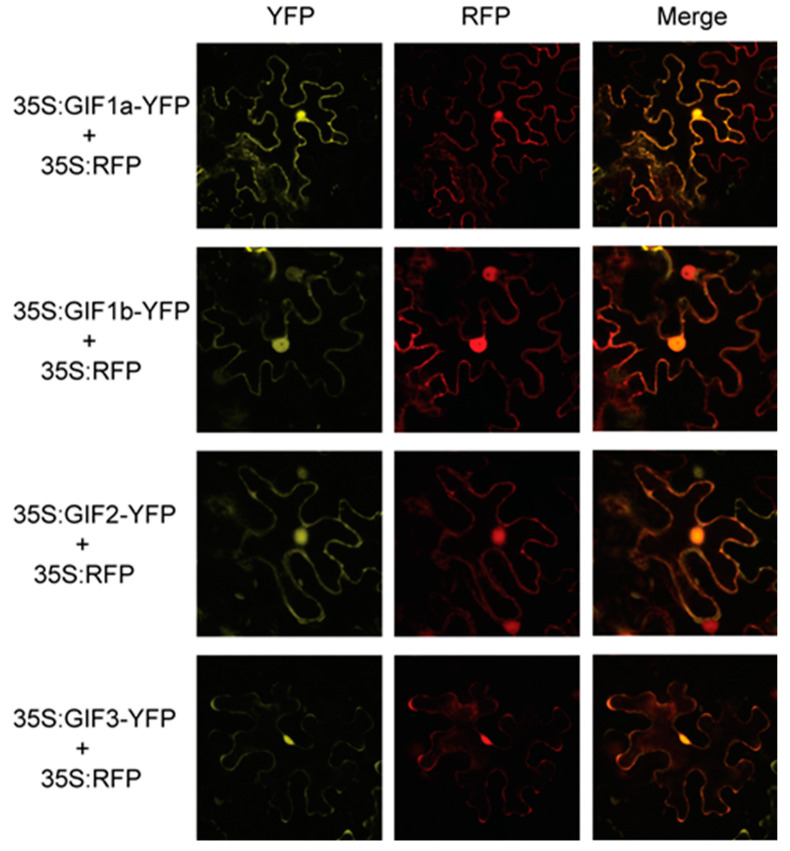
Subcellular localization of SlGIF– yellow fluorescent protein (YFP) fusion proteins. Tobacco leaves was infiltrated with *Argobacterium tumefaciens* (*A. tumefaciens*) containing a recombination vector (35S: GIFs-YFP) and a nuclear marker RFP (red fluorescent protein).

To further understand the functions of SlGIFs, we confirmed the subcellular localization of SlGIFs. The SlGIF:YFP fusion proteins were constructed under the control of the CaMV 35S promoter and expressed in the tobacco leaves. The confocal observation revealed fluorescence signals for all the SlGIF:YFP proteins in the nucleus and cytoplasm (Figure 6). Thus, all the SlGIF proteins were localized in the nucleus.

### 3.7. Interactions between the SlGIF Proteins and the SlGRF Proteins

**Figure 7:**
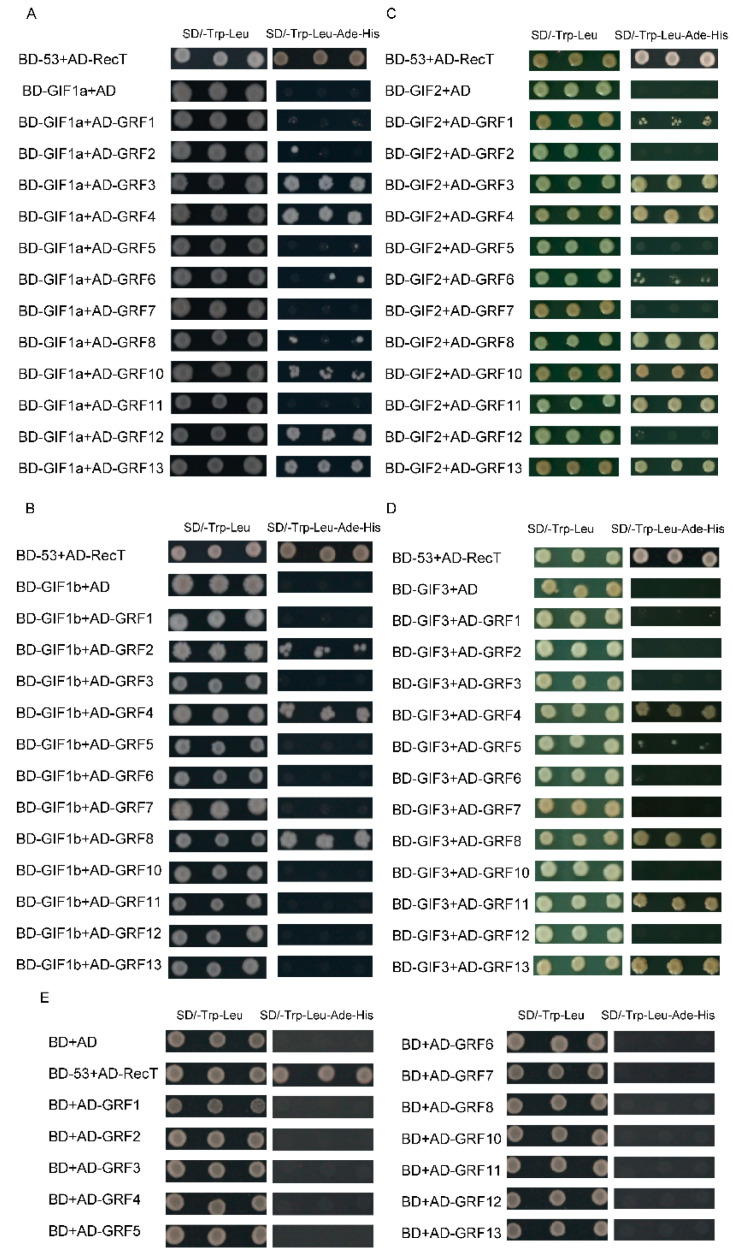
Interactions between GIF proteins and growth-regulating factors (GRF) proteins in a yeast two-hybrid assay. (**A**) GIF1a interacted with GRFs; (**B**) GIF1b interacted with GRFs; (**C**) GIF2 interacted with GRFs; (**D**) GIF3 interacted with GRFs; (**E**) the negative controls of GRFs. GIF proteins and GRF proteins were used as a bait and a prey, respectively, in different combinations. SD/-Trp-Leu was an SD medium lacking leucine and tryptophan. SD/-Trp-Leu-His-Ade was an SD medium lacking leucine, histidine, adenine, and tryptophan. BD was the pGBKT7 vector, and AD was the pGADT7 vector. Yeast cultures with transformed yeasts adjusted to have optical densities at 600 nm (OD_600_) of 1.0 and 2 μL yeast culture dilutions were spotted on SD/-Trp-Leu and SD/-Trp-Leu-His-Ade medium, respectively. The growth of the yeast strain on the SD/-Trp-Leu medium indicated that each pair of bait–prey was successfully transformed into AH109. The different growth conditions of the transformed yeasts on the SD/-Trp-Leu-His-Ade medium showed the strength of the interaction between the two proteins. Each group was performed for three repetitions.

GIFs proteins act as the co-activators of GRFs proteins in Arabidopsis, rice, and maize [10,12,14,15,16,18,19,25,28]. The interaction between the GIF proteins and the GRFs in tomatoes was assessed by a yeast two-hybrid assay. Four *GIF*s were cloned into pGBKT7 (bait vector), and 12 *GRF*s [47,48] were cloned into pGADT7 (prey vector) for an interaction assay. The four GIFs showed no self-activation activity, and each GIF proteins interacted with several GRF proteins (Figure 7 and Figure S4). SlGIF1a strongly interacted with SlGRF3, SlGRF4, SlGRF12, and SlGRF13 but weakly interacted with SlGRF10. Again, SlGIF1b strongly interacted with SlGRF4 and SlGRF8 but interacted weakly with SlGRF2. SlGIF2 strongly interacted with SlGRF3, SlGRF4, SlGRF8, SlGRF10, SlGRF11, and SlGRF13. However, it weakly interacted with SlGRF1 and SlGRF6 (Figure 7). SlGIF3 strongly interacted with SlGRF4, SlGRF8, SlGRF11, and SlGRF13 and weakly interacted with SlGRF5. SlGRF4 was the only GRF protein that interacted with the four *GIF* genes, indicating SlGRF4 may be involved in the functions of GIF proteins in tomatoes (Figure 7).

### 3.8. Relative Expression between SlGIF and SlGRF Genes and SlGIF Protein 

**Figure 8:**
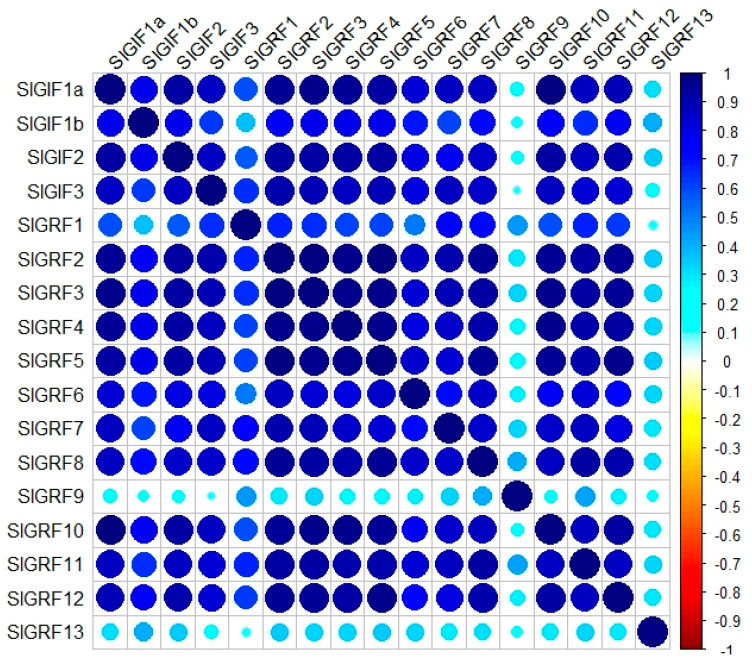
Co-expression between *GIF* and *GRF* genes in 536 tissues and samples. The sizes and colors of the circles indicate the values of the co-expression coefficients.

GIF1 interacts with GRF to regulate the expression of GRF in rice and Arabidopsis [7,8,9,15]. To further understand whether there is a regulatory relationship between the tomato *GIF* and *GRF* genes, the co-expression analyses between *SlGIFs* and *SlGRFs* were conducted. The expression profiles of *SlGIFs* and *SlGRFs* in different tissues were retrieved from the Tomato Functional Genomics Database (http://ted.bti.cornell.edu/). The expression levels of *GIF* and *GRF* genes from 536 samples (Table S3) in 18 transcriptome assays (Table S4) were employed for co-expression analysis between *SlGIFs* and *SlGRFs*. *SlGRF13*, *SlGRF9*, and *SlGRF1* showed lower co-expression levels between the *GIF* genes (Figure 8). Among the four *GIF* genes, *SlGIF1b* recorded a lower co-expression level between GRF genes. The expression levels of *SlGRF2*, *SlGRF3*, *SlGRF4* and *SlGRF5* were highly correlated with *SlGIF1a*, *SlGIF2*, and *SlGIF3*. In summary, the expression levels of *SlGIF1a*, *SlGIF2*, and *SlGIF3* had higher relationships with those of *SlGRF2*, *SlGRF3*, *SlGRF4* and *SlGRF5*, suggesting regulatory relationships between these genes.

**Figure 9:**
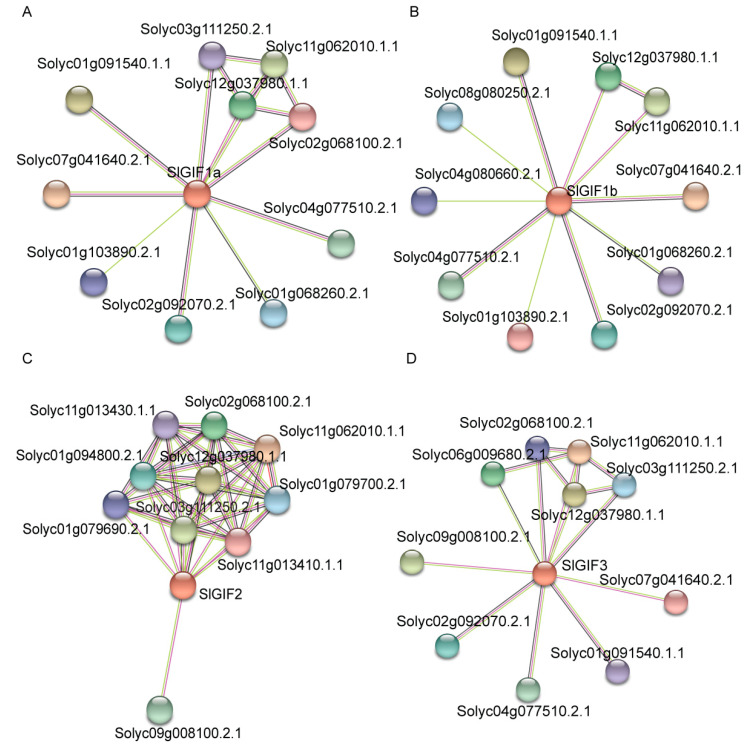
Predicted protein association networks analyses of SlGIF proteins: (**A**) SlGIF1a; (**B**) SlGIF1b; (**C**) SlGIF2; and (**D**) SlGIF3. The nodes represent the proteins, and the lines represent the protein–protein associations. Light blue and purple lines represent the known interactions from curated database or experimentally determined; green, red, and blue lines represent gene neighborhood, gene fusions, and gene co-occurrence, indicating the proteins are the predicted interactions; yellow, black, and light sky blue lines represent textiming, co-expression, and protein homology, respectively.

The STRING database (https://string-db.org/cgi/) was used to obtain putative protein–protein interaction among the SlGIF proteins and related proteins. Outputs from the STRING database were subsequently visualized in the standalone version of Cytoscape software [49] (Figure 9). Several proteins were predicted to associate with the SlGIF proteins, indicating diverse functions in growth and development. Among the proteins, two proteins (Solyc11g062010.1.1 and Solyc12g037980.1.1) were found to associate with all the SlGIF proteins. Based on the annotations of the proteins, Solyc11g062010.1.1 encoded a chromodomain helicase DNA-binding protein (CHD), related to chromatin remodeling [50,51,52], and Solyc12g037980.1.1 encoded an actin-like protein. 

## 4. Discussion

GIF proteins have been identified in several plants, such as *A. thaliana*, *O. sativa*, and *Z. mays.* They play essential roles in various biological processes [7,14,17,21,22,25,26,27,28,29,30]. However, there is limited study about the roles of *GIF* genes in tomatoes. In this study, the four *SlGIF* genes were identified in the tomato genome (Table 1). All four *SlGIF* genes showed different expression profiles in Heinz 1706 and LA1589 (Figure 4) and displayed different expression patterns in response to IAA and GA (Figure 5). The four SlGIF proteins were localized in the nucleus (Figure 6), interacted with various SlGRF proteins (Figure 7) and associated with the CHD protein and the actin-like protein (Figure 9). Additionally, SlGRF4 was a common protein that interacted with all four *SlGIF* proteins. Five SlGRF proteins and three SlGIF proteins showed higher co-expression relationships (Figure 8). Our results provide basic information of GIF proteins in tomatoes.

### 4.1. Phylogenetic Relationships and Structures of the SlGIF Gene Families

*GIF* genes were identified and distributed on four chromosomes in the tomato genome (Table 1). However, only three *GIF* genes were identified in Arabidopsis [14,19], rice [10], and maize [28], respectively. Gene duplications are one of significant forces driving the evolution of genomes and genetic systems in plants [53]. Our results indicated a gene duplication event occurred in the *GIF* gene family in tomatoes. Interestingly, two *GIF* genes (*SlGIF1a* and *SlGIF1b*) in tomatoes are the orthologous genes of *AtGIF1/AN3*, suggesting the duplication of the GIF1 in the tomato genome may have resulted in the expansion of the *SlGIF* genes. Remarkably, the GIF proteins in two monocots (rice and maize) and three dicots (Arabidopsis, potatoes, and tomatoes) clustered as orthologous pairs in a subgroup of each clade (OsGIF1 and ZmGIF1, OsGIF2 and ZmGIF2, and OsGIF3 and ZmGIF3) (Figure 1). The results implied the *GIF* gene family arose before monocots and dicots diverged. The four *SlGIF* genes possessed similar exon/intron structures (Figure 2A). However, AtGIF1/AN3 clustered in clade I had four exons, whereas AtGIF2 and AtGIF3 in clade II had five exons [29], indicating similar exon organization in Arabidopsis. The difference in the structures of the *GIF* genes in clade II between Arabidopsis and tomatoes implied varied functions of *GIF* genes may partly be ascribed to evolutionary divergence. 

### 4.2. Different Expression Patterns Shown by SlGIFs

In Arabidopsis, GIFs play essential roles in the development of leaves, male and female reproductive organs, cotyledons, and roots [17,20,21,22,24,29]. Our study indicated that *SlGIF1b* and *SlGIF2* had higher expressions in the early development of fruits, suggesting they play more important functions in early fruit development. Remarkably, cell division and expansion occurs in early fruit development which directly influence fruit weight and shape [54,55,56,57,58,59,60]. *GIF* genes play crucial roles in cell proliferation to determine fruit size [27,30,61]. For example, *an3* mutants generated in Arabidopsis involving *GIF* genes caused a decrease in cell number and slender-leaf phenotypes [19]. The rest of the triple mutants (*gif1*, *gif2*, and *gif3)* produced abnormal carpel margin meristem [17]. The *gif1* mutant in maize reduced indeterminate cells in leaf and stem, resulting in the production of narrow leaves and short internodes [28]. Enhancing the expression of *OsGIF1* led to increased sizes of multiple rice organs, such as stems, leaves, and grains [10,12,61]. Generally, GIF proteins may positively regulate fruit weight and size in tomatoes. 

### 4.3. Multifunctions in Tomatoes Played by the SlGIF Gene Family

The subcellular localization analysis indicated that SlGIF proteins were located in different organelles in a cell, including the nucleus, which is consistent with an earlier study [14]. The functional study showed that AtGIF1 proteins acted as transcriptional co-activators and interacted with AtGRF proteins in Arabidopsis [14,18,19,29], rice [10,12,16,25], and maize [28]. Thirteen (13) GRFs were identified in maize to interact with GIF1 [28]. AtGIF1 interacted with six GRF proteins in Arabidopsis [14,19,24], while OsGIF1 interacted with three GRF proteins [10,12,16]. These interactions suggested *GIF* genes play essential roles in complexes formed by GIF and GRF interactions. *GIF* genes may also mediate different pathways of plant growth and development via interacting with different *GRF* genes. All four SlGRFs interacted with SlGIFs in tomatoes (Figure 6), implying their multifunctions in tomatoes. 

Although *SlGIF1a* and *SlGIF1b* were the orthologous genes of *AtGIF1/AN3*, SlGIF1a and SlGIF1b interacted with different SlGRF proteins, except for the common protein SlGRF4. This indicated the functional divergence of SlGIF1a and SlGIF1b during evolution. Interestingly, SlGRF4 could interact with four SlGIF proteins in yeast. *AtGRF5* is an *SlGRF4* orthologous gene in tomatoes [48] and regulates cell proliferation in leaves [19]. These results implied the similar functions of *GIF* genes in cell proliferation in tomatoes by interacting with SlGRF4. This is inconsistent with the redundant functions of *GIF* genes in Arabidopsis [18]. Moreover, it has been reported that several GRF proteins were the downstream of the *GIF* genes in rice [25] and maize [27] and increasing the expression of *GIF* genes enhances the transcription levels of *GRF* genes. The different GRF and GIF functions in tomatoes may require further studies to unravel their specific functions. The co-expression analyses of *SlGIFs* and *SlGRFs* in tomatoes indicated the expression levels of *SlGRF2*, *SlGRF3*, *SlGRF4*, and *SlGRF5* had higher relationships with *SlGIF1a*, *SlGIF2*, and *SlGIF3*. The higher correlation of relative expression between the *SlGIF* and *SlGRF* genes showed that they may be regulated by the same TFs or the SlGRFs may function in the downstream functions of SlGIF1a, SlGIF2, and SlGIF3. The SlGIF proteins associated with the CHD protein and the actin-like protein as revealed by the protein association network analysis. This affirmed the primary function of *Sl*GIFs as co-activators [52]. This is consistent with the roles of AtGIF1/AN3 in Arabidopsis [26,27,62].

## 5. Conclusions

Four *GIF* genes were identified in the tomato genome. These genes are localized on four of the 12 tomato chromosomes. Our phylogenetic analysis classified the *GIF* genes into two major clades. The results from the conserved motifs, gene structure, and subcellular localization indicated that *SlGIF* genes contain SSXT motif and are localized in the nucleus and cytosol. A significant variation was recorded in the expression profiles of these genes at different stages of tomato growth and tissues under phytohormone treatments. We identified key *cis*-elements in the promoter regions, assessed expression profiles, protein–protein interaction and performed gene co-expression analyses to further evaluate the functions of *GIF*s in tomato growth and development. The identification and characterization of *GIF* gene family members in tomatoes provides a foundation for further functional studies for genetic improvement of tomatoes.

## Figures and Tables

**Table 1:** Physico-chemical characteristics of the growth-regulating factors-interacting factor (*GIF*) gene family in tomatoes.

Gene Name	Gene Loci	Chromosome Location (Strand)	aa	pIs/Mw
SlGIF1a	Solyc04g009820.2.1	SL2.50ch04:3139217-3143959 (+)	208	6.60/22.74 KDa
SlGIF1b	Solyc11g006230.1.1	SL2.50ch11:981174-984561 (−)	222	6.41/23.59 KDa
SlGIF2	Solyc03g082480.2.1	SL2.50ch03:45948144-45952630 (+)	199	5.85/21.83 KDa
SlGIF3	Solyc10g009280.2.1	SL2.50ch10:3267235-3271023 (−)	200	6.51/21.74 KDa

aa refers to protein length; pI refers to the theoretical isoelectric points; Mw refers to the molecular weight.

**Table 2:** Functionally described *cis*-elements identified in the promoters of the *SlGIF* genes.

*Cis*-Element	Members of GIFs	Functions of *Cis*-Element
ABRE	SlGIF1a, SlGIF1b, SlGIF2, and SiGIF3	*cis*-acting element involved in the abscisic acid responsiveness
ACA-motif	SlGIF3	part of gapA in (gapA-CMA1) involved with light responsiveness
ACE	SlGIF1a, SlGIF2, and SlGIF3	*cis*-acting element involved in light responsiveness
AE-box	SlGIF3	part of a module for light response
ARE	SlGIF1a, SlGIF1b, SlGIF2, and SiGIF3	*cis*-acting regulatory element essential for the anaerobic induction
AT1-motif	SlGIF1b	part of a light responsive module
ATCT-motif	SlGIF1b	part of a conserved DNA module involved in light responsiveness
AT-rich sequence	SlGIF1b and SlGIF3	element for maximal elicitor-mediated activation
AuxRR-core	SlGIF1a	*cis*-acting regulatory element involved in auxin responsiveness
Box 4	SlGIF1a, SlGIF2, and SlGIF3	part of a conserved DNA module involved in light responsiveness
CAAT-box	SlGIF1a, SlGIF1b, SlGIF2, and SiGIF3	common *cis*-acting element in promoter and enhancer regions
CAT-box	SlGIF1b and SlGIF2	*cis*-acting regulatory element related to meristem expression
CGTCA-motif	SlGIF1a and SlGIF2	*cis*-acting regulatory element involved in the MeJA-responsiveness
chs-CMA1a	SlGIF1b and SlGIF2	part of a light responsive element
circadian	SlGIF1a and SlGIF1b	*cis*-acting regulatory element involved in circadian control
GARE-motif	SlGIF1a and SlGIF2	gibberellin-responsive element
G-box	SlGIF1a, SlGIF1b, SlGIF2, and SiGIF3	*cis*-acting regulatory element involved in light responsiveness
GCN4_motif	SlGIF1b	*cis*-regulatory element involved in endosperm expression
GT1-motif	SlGIF1a and SlGIF2	Light-responsive element
LAMP-element	SlGIF1a	part of a light-responsive element
LAMP-element	SlGIF3	part of a light-responsive element
LTR	SlGIF1b, SlGIF2, and SlGIF3	*cis*-acting element involved in low-temperature responsiveness
MBS	SlGIF3	MYB-binding site involved in drought inducibility
MRE	SlGIF1a and SlGIF3	MYB-binding site involved in light responsiveness
O2-site	SlGIF1a and SlGIF1b	*cis*-acting regulatory element involved in zein metabolism regulation
Sp1	SlGIF1b	Light-responsive element
TATA-box	SlGIF1a, SlGIF1b, SlGIF2, and SiGIF3	core promoter element around –30 of transcription start
TATC-box	SlGIF1a	cis-acting element involved in gibberellin responsiveness
TCA-element	SlGIF1a, SlGIF1b, SlGIF2, and SiGIF3	*cis*-acting element involved in salicylic acid responsiveness
TC-rich repeats	SlGIF1a, SlGIF1b, and SlGIF3	*cis*-acting element involved in defense and stress responsiveness
TGA-box	SlGIF2	part of an auxin-responsive element
TGACG-motif	SlGIF1b and SlGIF3	*cis*-acting regulatory element involved in the MeJA responsiveness
TGA-element	SlGIF3	auxin-responsive element

## Supplementary Materials

Supplementary materials can be found at https://www.mdpi.com/2073-4425/11/12/1435/s1. Table S1: List of primers used in this study. Table S2: Protein sequences of GIFs from Arabidopsis, rice, maize, potatoes, and tomatoes. Table S3: Expression profiles of the *SlGIF* and *SlGRF* genes in 536 samples. Table S4: List of transcriptome analysis.
